# Differential role of pannexin-1/ATP/P2X_7_ axis in IL-1β release by human monocytes

**DOI:** 10.1096/fj.201600256

**Published:** 2017-02-28

**Authors:** Katarzyna Parzych, Anna V. Zetterqvist, William R. Wright, Nicholas S. Kirkby, Jane A. Mitchell, Mark J. Paul-Clark

**Affiliations:** * Department of Cardiovascular Pharmacology, Vascular Biology, National Heart and Lung Institute, Imperial College London, London, United Kingdom;; † Department of Clinical Sciences, Lund University, Malmö, Sweden

**Keywords:** inflammasome, innate immunity, Toll-like receptors, potassium channels

## Abstract

IL-1β release is integral to the innate immune system. The release of mature IL-1β depends on 2 regulated events: the *de novo* induction of pro-IL-1β, generally *via* NF-κB-dependent transduction pathways; and the assembly and activation of the NLRP3 inflammasome. This latter step is reliant on active caspase-1, pannexin-1, and P2X_7_ receptor activation. Pathogen-associated molecular patterns in gram-positive and gram-negative bacteria activate IL-1β release from immune cells *via* TLR2 and TLR4 receptors, respectively. We found that pro-IL-1β and mature IL-1β release from human monocytes is stimulated by the TLR2 agonists Pam_3_CSK4 or FSL-1, as well as the TLR4 agonist LPS in the absence of additional ATP. TLR2 agonists required pannexin-1 and P2X_7_ receptor activation to stimulate IL-1β release. In contrast, IL-1β release stimulated by the TLR4 agonist LPS is independent of both pannexin-1 and P2X_7_ activation. In the absence of exogenous ATP, P2X_7_ activation requires endogenous ATP release, which occurs in some cells *via* pannexin-1. In line with this, we found that LPS-stimulated human monocytes released relatively low levels of ATP, whereas cells stimulated with TLR2 agonists released high levels of ATP. These findings suggest that in human monocytes, both TLR2 and TLR4 signaling induce pro-IL-1β expression, but the mechanism by which they activate caspase-1 diverges at the level of the pannexin-1/ATP/P2X_7_ axis.—Parzych, K., Zetterqvist, A. V., Wright, W. R., Kirkby, N. S., Mitchell, J. A., Paul-Clark, M. J. Differential role of pannexin-1/ATP/P2X_7_ axis in IL-1β release by human monocytes.

IL-1β is a proinflammatory cytokine with a pivotal role in innate immunity and is a monotherapeutic target for a number of inflammatory conditions, such as gout (1). Unlike other proinflammatory cytokines, such as TNF-α and IL-6, IL-1β is not processed in the traditional manner through the Golgi apparatus. Instead, it is cleaved from its precursor molecule pro-IL-1β. In this fashion, mature IL-1β production requires 2 distinct steps: first a priming stage where pro-IL-1β is induced, followed by the assembly and activation of the NLRP3 inflammasome, which enzymatically cleaves caspase-1 to its active form.

The priming step in IL-1β production is triggered by many proinflammatory stimuli, including bacterial pathogen-associated molecular patterns, which activate pattern recognition receptors (2), such as the TLRs. Production of mature IL-1β from pro-IL-1β is less well understood, particularly the processes leading to activation of the NLRP3 inflammasome, which seem to vary between cell types and particular stimuli. Current knowledge indicates that NLRP3 inflammasome activation is mediated by pannexin-1, a large-pore channel that facilitates the release of ATP and potassium ions. ATP released *via* pannexin-1 activates the purine receptor P2X_7_, resulting in additional potassium ion efflux (3). ATP/P2X_7_ signaling can then further activate pannexin-1, resulting in an amplification loop for potassium efflux and cellular hyperpolarization (4). This triggers nucleotide-binding oligomerization domain inflammasome assembly and activation.

Potassium efflux and cell hyperpolarization facilitated by the pannexin-1/ATP/P2X_7_ axis is probably the best-studied mechanism by which NLRP3 inflammasome and caspase-1 activation occurs. There are, however, pannexin-1-independent pathways that lead to NLRP3 inflammasome. For example, physical perturbation of the cell membrane by nanoparticles or crystals of cholesterol or monosodium urate can cause hyperpolarization of cells through direct membrane damage (5–7).

In many studies designed to investigate IL-1β release from cells, the assembly and activation step in the process is artificially modeled by simply adding very high levels of ATP, in the millimolar range (8). This has proved useful in determining aspects of cell priming and the function of pannexin-1 and P2X_7_. However, exogenous ATP in millimolar concentrations is unlikely to occur in the body, even at sites of inflammation. Thus, understanding how IL-1β release occurs in more physiologic settings requires the inclusion of protocols in which endogenous pathways are compared without an artificially large exogenous ATP challenge (9).

Monocytes release ATP when stimulated with LPS (10), while human macrophages and dendritic cells differ from monocytes in their ability to release both ATP and mature IL-1β after stimulation with TLR ligands. For example, human monocytes, but not macrophages or dendritic cells, have been shown to have constitutively active caspase-1, meaning that they release mature IL-1β after stimulation with TLR2 or TLR4 agonists, without the requirement of exogenous ATP (9). However, the role of pannexin-1 and/or P2X_7_ in endogenous release of IL-1β stimulated by TLR4 *vs.* TLR2 agonists has not been addressed.

Thus, in the present study, we compared the relative ability of TLR2 and TLR4 agonists to activate caspase-1, express pro-IL-1β, and release ATP and mature IL-1β from human monocytic cells. We also determined the relative role that pannexin-1 and P2X_7_ may have in IL-1β production induced by TLR2 *vs.* TLR4.

## MATERIALS AND METHODS

### Cell culture

Human acute monocytic leukemia cell line THP-1 was obtained from the European Collection of Cell Cultures (Salisbury, United Kingdom) and cultured in RPMI 1640 supplemented with 10% filtered heat-inactivated fetal calf serum, 2 mM glutamine, and 100 U/ml penicillin/streptomycin and maintained at 37°C containing 5% CO_2_. Cells were seeded in 96-well plates at 1 × 10^5^ cells per well for 12 h before treatment. Cell viability was assessed using alamarBlue (Thermo Fisher Scientific, Waltham, MA, USA) after all treatments, and IL-1β and pro-IL-1β release was measured by ELISA (R&D Systems, Minneapolis, MN, USA).

### Inhibitor treatments

Human monocytes were treated with TLR4 agonist LPS (0.001 to 1 μg/ml), or TLR2 agonists Pam_3_CSK4 (0.001 to 1 μg/ml) and/or FSL-1 (0.001 to 1 μg/ml) for 3 or 24 h. In some experiments, cells were pulsed for 30 min with ATP (1–5 mM) after activation. In the inhibition studies, cells were pretreated for 30 min with inhibitors of caspase-1 (Z-VAD-FMK; 0.01 to 1 µg/ml), pannexin-1 (carbenoxolone; 0.03 to 30 µg/ml), P2X_7_ (AZ11645373; 0.01 to 1 µg/ml), or MaxiK (large-conductance, voltage-dependent, and Ca^2+^-activated K^+^) channel (paxilline 5–20 µM, or TEA 5–20 mM), after which they were stimulated with LPS, Pam_3_CSK4, or FSL-1.

### Small interfering RNA transfection

THP-1 cells were resuspended in solution V (VCA-1003). siRNA was added (20–100 nM), and cells were electroporated using standard methods (Amaxa Nucleofector transfection system; Lonza, Basel, Switzerland). After incubation in prewarmed medium for 10 min at 37°C, cells were transferred onto a 96-well plate. After 48 h, cells were treated as above. Small interfering RNA (siRNA) sequences used were AllStars Negative Control siRNA (Qiagen, Germantown, MD, USA) and On-TargetPlus SmartPool, Human Panx1 (GE Dharmacon, Lafayette, CO, USA), as follows: 5′-UAAGUGAGGUCAAGUCAUA-3′, 5′-CGGCAGAGCUCCAAGGUAU-3′, 5′-CAUAUUUGCUCAGACUUGA-3′, and 5′-CACUGUGGCUGCAUAAGUU-3′.

### Western blot analysis

Western blot analysis was performed 48 h after gene knockdown. Whole-cell protein extracts were prepared on ice using a lysis buffer containing 50 mM HEPES pH 7.5, 50 mM sodium fluoride, 5 mM sodium pyrophosphate, 1 mM EDTA, 1 mM DTT, 10% glycerol, 1% Triton X-100, and complete EDTA-free Protease Inhibitor Cocktail (Roche, Basel, Switzerland). Protein extracts were loaded on a SDS-PAGE gel, transferred to PVDF membrane, and subjected to the incubation with individual antibodies. Primary antibodies used were as follows: pannexin-1 (Abcam, Cambridge, MA, USA), P2X_7_ (Alomone Labs, Jerusalem, Israel), β-tubulin (Cell Signaling Technology, Danvers, MA, USA). The PageRuler Plus Prestained Protein Ladder (Pierce, Rockford, IL, USA) was used as a molecular weight marker.

### Caspase-1 activity

Caspase-1 activity was measured by FAM FLICA with an *in vitro* caspase detection kit according to the manufacturer's instructions (Abcam). Briefly, A549 cells were treated with either DMSO or LPS (0.1 μg/ml) or Pam_3_CSK4 (0.1 μg/ml) for 24 h. Cells were labeled with FAM FLICA for 60 min at 37°C. After washing, cells were read on a fluorescent plate reader (excitation wavelength 488 nm, emission wavelength 530 nm).

### ATP release

ATP release from THP-1 cells was measured using the ChronoLume luciferase assay system (Chrono-Log, Havertown, PA, USA). THP-1 cells, cultured in 96-well microtiter plates, were stimulated with LPS (0.1 μg/ml) or Pam_3_CSK4 (0.1 μg/ml). Cells were incubated for 30 min; then ChronoLUME reagent containing firefly luciferase (16 μg/ml final) and d-luciferin (1760 U/ml final) was added to each well. After brief mixing, the luminescence read using a Mithras LB940 multimode plate reader (Berthold Technologies, Bad Wildbad, Germany). ATP release was calculated with reference to the luminescence of wells containing ChronoLume and 40 μM exogenous ATP standard (Chrono-Log).

## RESULTS AND DISCUSSION

**Figure 1.**
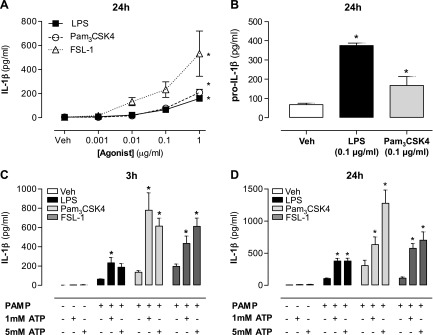
Production of pro-IL-1β and release of mature IL-1β from human monocytes in response to gram-positive and gram-negative pathogen-associated molecular patterns. *A*) THP-1 monocytes were treated with concentration responses (0.001 to 1 μg/ml) to LPS, Pam_3_CSK4, and FSL-1 for 24 h, and IL-1β release was measured by ELISA. *B*) Intracellular levels of pro-IL-1β were measured by ELISA after 24 h stimulation with LPS (0.1 μg/ml) and Pam_3_CSK4 (0.1 μg/ml). *C*, *D*) In addition, monocytes were treated with LPS (1 µg/ml) or Pam_3_CSK4 (1 µg/ml) for 3 (*C*) or 24 h (*D*), after which medium was removed and cells were pulsed for 30 min with either ATP (5 mM) or fresh medium. IL-1β release was measured after 3 and 24 h. Data represent means ± sem of total of at least 9 replicates. *P ≤ 0.05 as assessed by 1-way ANOVA followed by Dunnett’s *post hoc* test.

To assess the effects of TLR2 and TLR4 ligands on the release of IL-1β by human monocytes, cells were treated for 24 h with FSL-1 or Pam_3_CSK4 (TLR2), or with LPS (TLR4). In each case, Pam_3_CSK4, FSL-1, or LPS caused a concentration-dependent release of mature IL-1β into the conditioned media without the requirement of exogenous ATP (**Fig. 1*A***). In line with this, both Pam_3_CSK4 and LPS increased the release of unprocessed pro-IL-β (Fig. 1*B*). As expected, and as others have shown, when cells were primed with Pam_3_CSK4, FSL-1, or LPS and challenged with a brief (20 min) exposure to millimolar concentrations of exogenous ATP, mature IL-1β was released (Fig. 1*C, D*). IL-1β release in response to exogenous ATP is known to require pannexin-1 and P2X_7_ cooperation (11). However, in the absence of exogenous ATP, the role these pathways play in TLR4-mediated *vs.* TLR2-mediated IL-1β release is incompletely understood.

**Figure 2.**
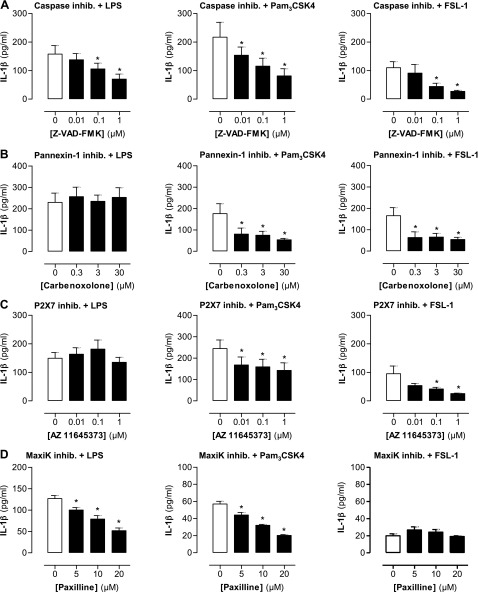
Effect of inhibiting inflammasome components on TLR2- and TLR4-induced IL-1β release from human monocytes. THP-1 monocytes were stimulated for 24 h with LPS (0.1 μg/ml), Pam_3_CSK4 (0.1 μg/ml), and FSL-1 (0.1 μg/ml) after 30 min pretreatment with pan-caspase inhibitor Z-VAD-FMK (0.01 to 1 μM) (*A*), pannexin-1 inhibitor carbenoxolone (0.3–30 μM) (*B*), P2X_7_ inhibitor AZ11645373 (0.01 to 10 μM) (*C*), and MaxiK channel inhibitor paxilline (5–20 μM) (*D*), and IL-1β levels were measured by ELISA. Data represent means ± sem of total of at least 9 replicates. *P ≤ 0.05 as assessed by 1-way ANOVA followed by Dunnett’s *post hoc* test.

The processing of IL-1β from pro-IL-1β requires caspase-1 (12). Thus, as expected, we found that IL-1β release stimulated by either LPS or Pam_3_CSK4 and FSL-1 was inhibited in a concentration-dependent manner by the pan-caspase inhibitor Z-VAD-FMK (**Fig. 2*A***). However, while IL-1β release stimulated by the TLR2 ligands Pam_3_CSK4 and FSL-1 was inhibited by the pannexin-1 inhibitor carbenoxolone, release stimulated by the TLR4 agonist LPS was completely unaffected (Fig. 2*B*). Activation of pannexin-1 results in ATP release and subsequent activation of P2X_7_ (13). In line with what we observed with pannexin-1, inhibition of P2X_7_ using pharmacologic concentrations of AZ11645373 inhibited IL-β release by Pam_3_CSK4 and FSL-1, but did not affect release by LPS (Fig. 2*C*). It has been shown in human macrophages that the large conductance potassium channel MaxiK plays a crucial role in the cellular activation by TLRs (14). This may therefore be an alternative way for potassium efflux to occur in human monocytes, leading to the consequent assembly and activation of the NLRP3 inflammasome after TLR4 stimulation. Impeding this process by using the MaxiK inhibitor paxilline resulted in an inhibition of IL-1β from both LPS- and Pam_3_CSK4-stimulated cells (Fig. 2*D*), thus suggesting that this pathway plays an important role in both TLR2 and TLR4 activation of the NLRP3 inflammasome.

**Figure 3.**
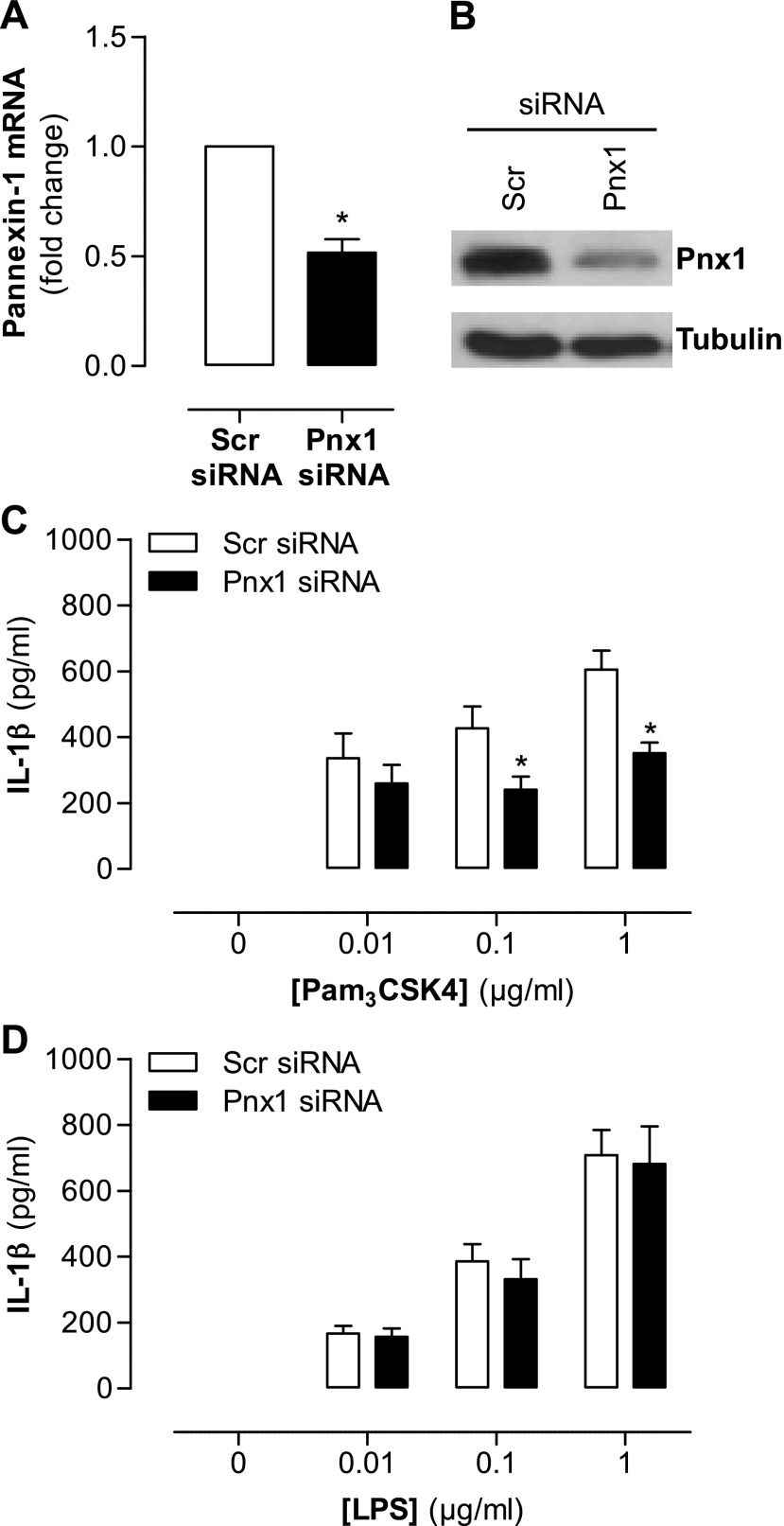
Effect of pannexin-1 gene knockdown on TLR2- and TLR4-induced IL-1β release from human monocytes. THP-1 cells were transfected with 100 µM of pannexin-1 siRNA or scrambled siRNA using Amaxa Nucleofector transfection system. *A*, *B*) After 48 h, gene expression was measured by real-time quantitative PCR (*A*) and protein levels were measured by Western blot analysis (*B*). *C*, *D*) Transfected THP-1 monocytes were stimulated with Pam_3_CSK4 (0.01 to 1 μg/ml) (*C*) and LPS (0.01 to 1 μg/ml) (*D*). PCR transfection data are representative of 3 separate transfections; Western blot analysis is representative of these transfections. Stimulation data represent means ± sem of 9 replicates. *P ≤ 0.05 as assessed by 1-way ANOVA followed by Bonferroni *post hoc* test.

Using pharmacologic tools to dissect signaling pathways can be subject to compromise by nonspecific drug effects. AZ11645373 is a well-validated drug and is highly specific to human P2X_7_ receptors when used at mid–high nanomolar concentrations (15). Carbenoxolone, while widely used as a pannexin-1 inhibitor, is not specific and has well-documented effects on other biologic pathways (16). Thus, in order to validate our observation that TLR4-induced IL-1β production occurs independently of pannexin-1 and P2X_7_, we performed gene knockdown of pannexin-1. Selected siRNA sequences for pannexin-1 reduced its expression at both the transcriptional and translation level (**Fig. 3*A, B***), and, directly corroborating our data using pharmacologic inhibitors, it also reduced IL-1β release induced by the TLR2 agonist Pam_3_CSK4 (Fig. 3*C*) but not by the TLR4 agonist LPS (Fig. 3*D*). By way of a functional control, production of CXCL8, which is stimulated by TLR2 or TLR4 agonists in parallel with IL-1β and independently of caspase-1, was not affected by any of the inhibitor drugs (Supplemental Fig. 1). Similarly, cell viability was not compromised in any of the protocols used (Supplemental Fig. 2).

**Figure 4.**
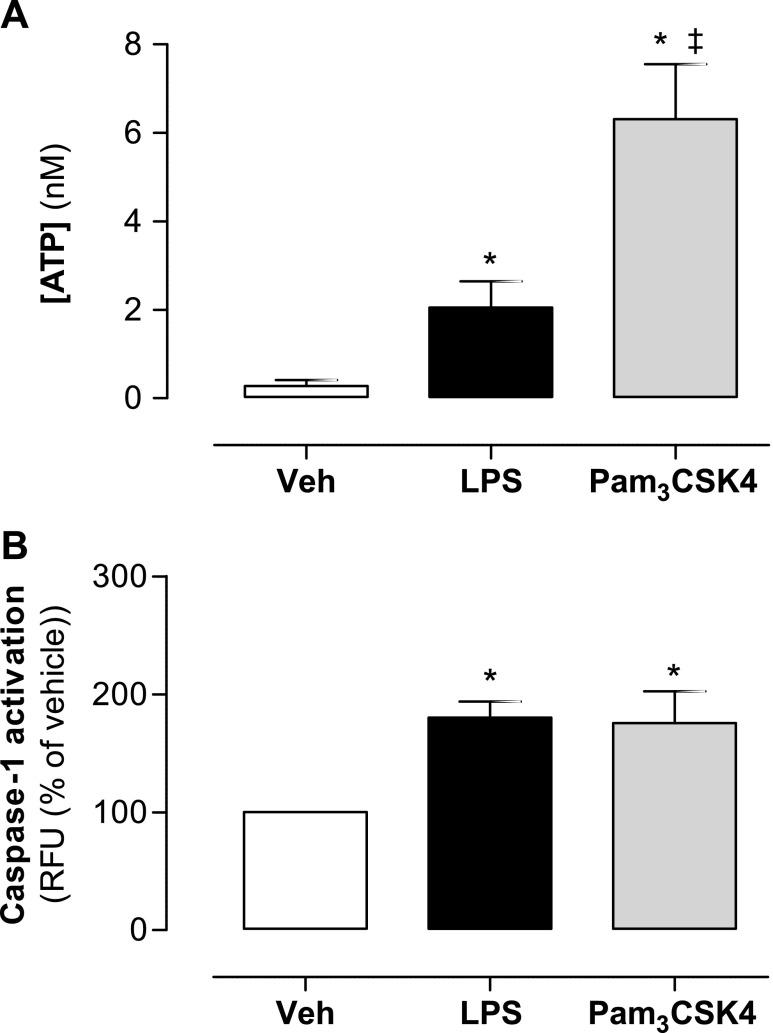
ATP release and caspase-1 activity in human monocytes stimulated with TLR2 and TLR4 ligands. THP-1 monocytes were stimulated with LPS (0.1 μg/ml) and Pam_3_CSK4 (0.1 μg/ml). ATP release was assessed after 30 min (*A*) and caspase-1 activity after 24 h (*B*). Data represent means ± sem of 9 replicates. *P ≤ 0.05 as assessed by 1-way ANOVA followed by Dunnett’s *post hoc* test.

Pannexin-1 activation and gap junction formation are recognized mechanisms by which endogenous ATP is actively released from cells (9). Once released from cells primed for pro-IL-1β induction, ATP acts on P2X_7_ receptors to amplify potassium ion efflux (17). Because our data suggest that TLR2, but not TLR4, receptor activation involves pannexin-1, we measured release of ATP from cells stimulated with LPS *vs.* Pam_3_CSK4. We found that monocytes stimulated with Pam_3_CSK4 released significantly higher levels of ATP than monocytes stimulated with LPS (**Fig. 4*A***). These observations, together with our data implicating the involvement of P2X_7_ in TLR2-mediated, but not TLR4-mediated, IL-1β release, suggest that LPS-induced ATP release from monocytes is not sufficient to initiate NLRP3 inflammasome assembly, whereas levels of ATP released by Pam_3_CSK4 constitute a pharmacologically relevant event.

The function of pannexin-1 and P2X_7_ in IL-1β release is ultimately to mediate potassium ion efflux; this is required for assembly and activation of the NLRP3 inflammasome and subsequent activation of caspase-1. Netea *et al.* (9) showed that in primary monocytes, but not in THP-1 cells, caspase-1 is constitutively active. Thus, we next sought to determine whether TLR4 signaling with LPS differed from TLR2 signaling with Pam_3_CSK4 at the level of caspase-1 activation. We found that caspase-1 was activated at identical levels when cells were treated with either Pam_3_CSK4 or LPS (Fig. 4*B*), thus suggesting that the extent of NLRP3 activation, and hence IL-11β release, is similar with both TLR2 and TLR4 activation. However, it seems that the monocytes have different ways of processing these stimuli.

**Figure 5.**
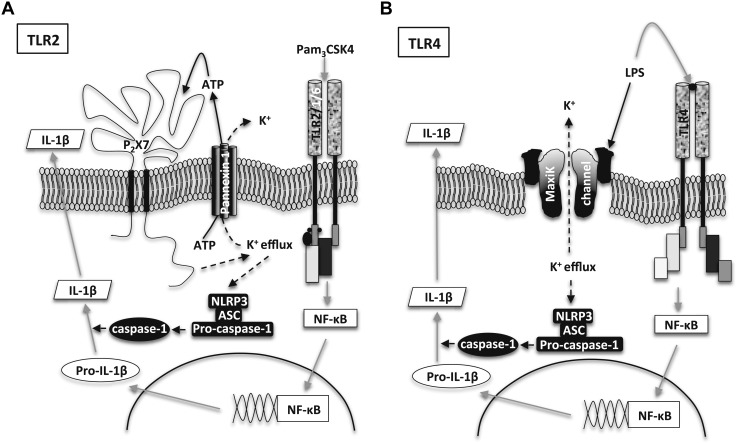
Putative signaling for pannexin-1/ATP/P2X_7_-dependent and -independent release of IL-1β by TLR2 and TLR4 agonists, respectively. IL-1β release was induced by TLR2 and TLR4 ligands from human monocytes. *A*) TLR2 agonists induce expression of pro-IL-1β *via* NF-κB and activate ATP release *via* panexin-1. ATP activates P2X_7_, which mediates potassium ion efflux and hyperpolarization, leading to activation of caspase-1, which cleaves pro-Il-1β, resulting in release of mature IL-1β. *B*) TLR4 agonist LPS activates TLR4 and NF-κB pathways, resulting in increased expression of pro-IL-1β. LPS evades pannexin-1 activation resulting in reduced ATP release, but activates MaxiK channels resulting in potassium ion efflux and hyperpolarization, and thus resulting in release of mature IL-1β.

Taken together, our observations, using a simple monocytic cell line model, show that TLR2- and TLR4-mediated activation of the NLRP3 inflammasome and subsequent activation of caspase-1 can occur by completely different signaling pathways. For TLR2, we have defined these pathways and hypothesize that the sequence of events is *1*) activation of pannexin-1, *2*) release of ATP, *3*) activation of P2X_7_, *4*) efflux of potassium ions, and *5*) activation of NLRP3 (**Fig. 5*A***). For TLR4, a full characterization of the steps leading to activation of the NLRP3 inflammasome and caspase-1 was beyond the scope of this study, but our results indicated that it was independent of either pannexin-1 or P2X_7_. Possible pathways include the activation of MaxiK channels, which are activated by LPS (18, 19) and facilitate potassium efflux without involvement of the pannexin-1/ATP/P2X_7_ axis (Fig. 5*B*).

## Supplementary Material

Supplemental Data

## Abbreviations

MaxiK: large-conductance, voltage-dependent, and Ca^2+^-activated K^+^
siRNA: small interfering RNA
