# Supplementary material for: Differential role of pannexin-1/ATP/P2X7 axis in IL-1β release by human monocytes
Source: FASEB J. 2017 Feb 28;31(6):2439–45. doi: 10.1096/fj.201600256 (PMC5507675; doi:10.1096/fj.201600256)
Supplement: Supplemental Data [file supp_31_6_2439__index.html]

Differential role of pannexin-1/ATP/P2X7 axis in IL-1β release by human monocytes — Supplemental Data 

# Differential role of pannexin-1/ATP/P2X7 axis in IL-1β release by human monocytes

## Supplemental Data

- Supplemental Data
- Supplemental Data
