# Supplementary material for: Differential role of pannexin-1/ATP/P2X7 axis in IL-1β release by human monocytes
Source: FASEB J. 2017 Feb 28;31(6):2439–45. doi: 10.1096/fj.201600256 (PMC5507675; doi:10.1096/fj.201600256)
Supplement: Supplemental Data [file supp_fj.201600256_Supplemental_Data1.docx]

**Supplementary Figure legends**

**Supplementary Figure 1. The effect inhibiting inflammasome components on TLR2 and TLR4 induced CXCL8 release from human monocytes.** THP-1 monocytes were stimulated for 24h with LPS (0.1μg/ml), Pam_3_CSK4 (0.1μg/ml) and FSL-1 (0.1μg/ml) after 30min pre-treatment with (A) pan-caspases inhibitor Z-VAD-FMK (0.01-1μM), (B) pannexin-1 inhibitor carbenoxolone (0.3-30μM) and (C) P2X_7_ inhibitor AZ 11645373 (0.01-10μM) and CXCL8 realease measure by ELISA. Data represents mean ± SEM of a total of at least n=9 replicates. *Denotes p≤ 0.05 as assessed using a one-way ANOVA followed by a Dunnett’s post-hoc test.

**Supplementary Figure 2. Cell viability data for pharmacological interventions and siRNA gene knockdown studies.** THP-1 monocytes were stimulated for 24h with LPS (0.1μg/ml), Pam_3_CSK4 (0.1μg/ml) and FSL-1 (0.1μg/ml) after 30min pre-treatment with (A) pan-caspases inhibitor Z-VAD-FMK (0.01-1μM), (B) pannexin-1 inhibitor carbenoxolone (0.3-30μM) and (C) P2X_7_ inhibitor AZ 11645373 (0.01-10μM) (D) MaxiK inhibitor paxilline and cell viability assessed using Alamar blue. (E) The effect of siRNA gene knockdown on cell viability was assessed after 48h using Alamar blue. Data represents mean ± SEM of a total of at least n=3 replicates. *Denotes p≤ 0.05 as assessed using a one-way ANOVA followed by a Dunnett’s post-hoc test.
