# Supplementary material for: Differential role of pannexin-1/ATP/P2X7 axis in IL-1β release by human monocytes
Source: FASEB J. 2017 Feb 28;31(6):2439–45. doi: 10.1096/fj.201600256 (PMC5507675; doi:10.1096/fj.201600256)
Supplement: Supplemental Data [file supp_fj.201600256_Supplemental_Figure2.pptx]

## Slide 1
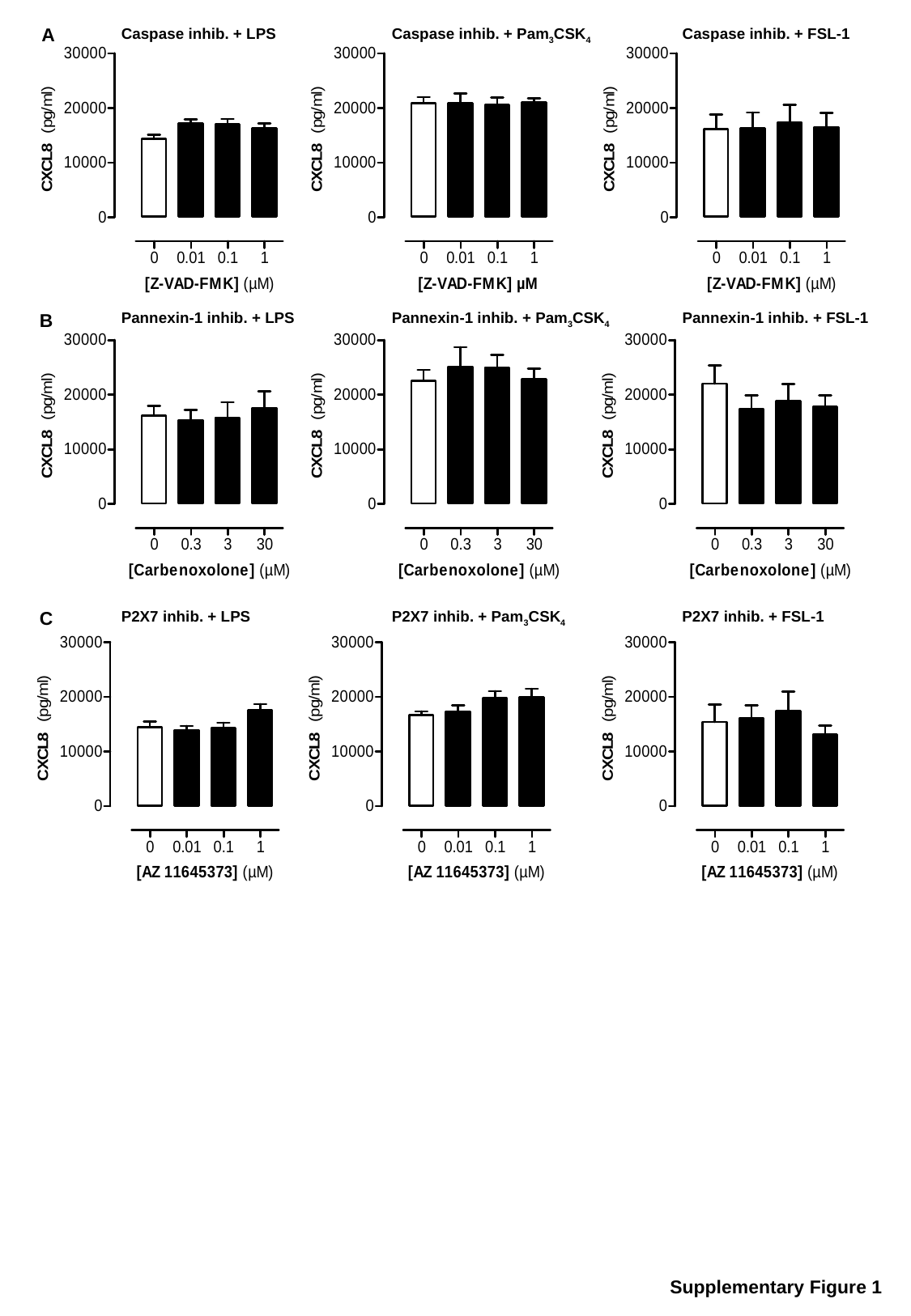

A
Caspase inhib. + LPS
Caspase inhib. + Pam3CSK4
Caspase inhib. + FSL-1
B
Pannexin-1 inhib. + LPS
Pannexin-1 inhib. + Pam3CSK4
Pannexin-1 inhib. + FSL-1
C
P2X7 inhib. + LPS
P2X7 inhib. + Pam3CSK4
P2X7 inhib. + FSL-1
Supplementary Figure 1

## Slide 2
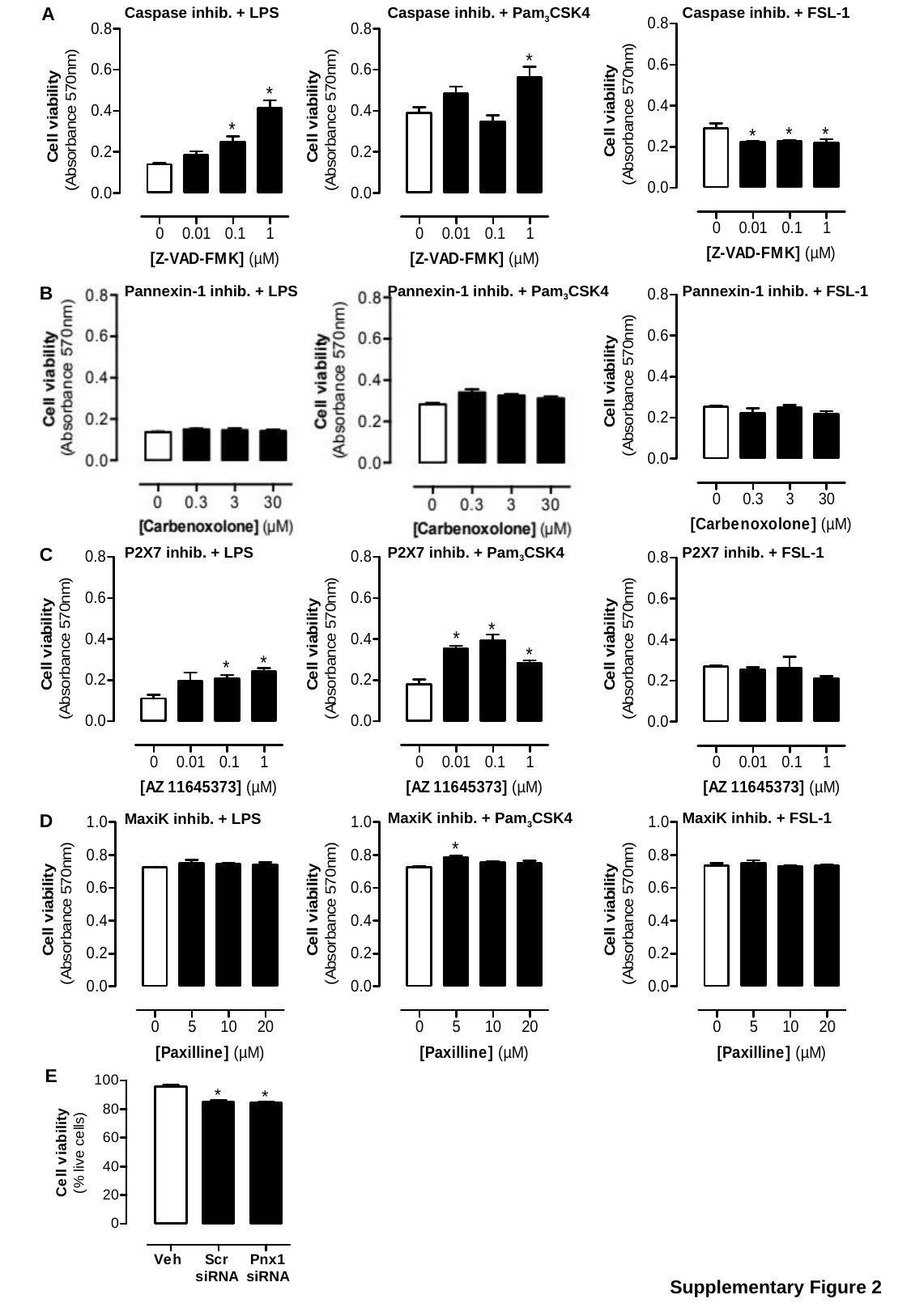

A
Caspase inhib. + LPS
Caspase inhib. + Pam3CSK4
Caspase inhib. + FSL-1
B
Pannexin-1 inhib. + LPS
Pannexin-1 inhib. + Pam3CSK4
Pannexin-1 inhib. + FSL-1
C
P2X7 inhib. + LPS
P2X7 inhib. + Pam3CSK4
P2X7 inhib. + FSL-1
MaxiK inhib. + Pam3CSK4
MaxiK inhib. + FSL-1
D
MaxiK inhib. + LPS
E
Supplementary Figure 2
